# An Immunogenic Cell Death-Related Classification Predicts Prognosis and Response to Immunotherapy in Head and Neck Squamous Cell Carcinoma

**DOI:** 10.3389/fimmu.2021.781466

**Published:** 2021-11-19

**Authors:** Xinwen Wang, Shouwu Wu, Feng Liu, Dianshan Ke, Xinwu Wang, Dinglong Pan, Weifeng Xu, Ling Zhou, Weidong He

**Affiliations:** ^1^ Department of Orthopedics, The People’s Hospital of Jiangmen, Jiangmen, China; ^2^ Department of Otolaryngology, Quanzhou First Hospital Affiliated to Fujian Medical University, Quanzhou, China; ^3^ Department of Orthopedics, Dongguan People’s Hospital, Dongguan, China; ^4^ Department of Orthopedics, The First Hospital of Putian City, Putian, China; ^5^ Department of Radiation Oncology, The Second Affiliated Hospital of Fujian Medical University, Quanzhou, China; ^6^ Department of Medical Oncology, The Affiliated Cancer Hospital of Zhengzhou University, Zhengzhou, China; ^7^ Department of Radiation Oncology, Dongguan People’s Hospital, Dongguan, China

**Keywords:** immunogenic cell death, head and neck squamous cell carcinoma, prognosis, tumor microenvionment, immunotherapy

## Abstract

Immunogenic cell death (ICD) has been classified as a form of regulated cell death (RCD) that is sufficient to activate an adaptive immune response. Accumulating evidence has demonstrated the ability of ICD to reshape the tumor immune microenvironment through the emission of danger signals or DAMPs, which may contribute to the immunotherapy. Currently, identification of ICD-associated biomarkers that stratify patients according to their benefit from ICD immunotherapy would be of great advantage. Here, we identified two ICD-associated subtypes by consensus clustering. ICD-high subtype was associated with the favorable clinical outcomes, abundant immune cell infiltration, and high activity of immune response signaling. Besides, we established and validated an ICD-related prognostic model that predicted the survival of HNSCC and was associated with tumor immune microenvironment. In conclusion, we established a new classification system of HNSCC based on ICD signatures. This stratification had significant clinical outcomes for estimating prognosis, as well as the immunotherapy of HNSCC patients

## Introduction

Immunogenic cell death has been identified as a type of regulated cell death (RCD) that is enough to trigger an adaptive immune response (1, 2). Numerous comprehensive research investigations have been conducted in the past few years to explore the notion of immunogenic cell death. Damage-associated molecular patterns (DAMPs), including released high mobility group protein B1 (HMGB1), secreted ATP, and surface-exposed calreticulin (CRT) are primarily responsible for ICD’s immunogenic properties (3). The concept of cancer immunotherapy is to harness the immune system in triggering an antitumor immune response. The capacity of ICD to trigger certain anticancer immune responses has been strongly emphasized by growing research (4). Notable, while ICD has been used in a number of preclinical models, the existing evidence regarding the application of ICD in clinical practice is not sufficiently convincing (5). Thus, further study in patients ought to be carried out in a clinical context to evaluate the possibilities of ICD. In particular, discovering biomarkers that categorize patients premised on their response with ICD immunotherapy would be extremely beneficial.

Head and neck squamous cell carcinoma (HNSCC) has a dismal prognosis, with relatively low rate of survival in the case of late-stage tumor (6). Over the last few decades, limited improvements have occurred in terms of survival trends. An increasing amount of research evidence suggests that the immune system performs an instrumental function in the development of HNSCC since tumor cells elude immunosurveillance *via* activating inhibitory checkpoint pathways that inhibit anti-tumor T-cell responses (7). Immunotherapy (such as monoclonal antibodies, immune checkpoint inhibitors, costimulatory agonists, cancer vaccine, etc.) is demonstrated as an auspicious strategy for treating HNSCC patients (8, 9). The continued development of cancer immunotherapy and greater knowledge of the responses of T cells to targeted immune checkpoint treatments, in conjunction with the effectiveness of clinical studies of medicines blocking these immune checkpoints, will result in more prominent investigation forecasting and recognizing accurate biomarkers of immunotherapy in HNSCC.

In this study, we aimed to identify ICD associated biomarkers and develop an ICD risk model that predicts the immune microenvironment, prognosis, and response to immunotherapy in HNSCC. In the future, this technique can help physicians to make significant judgments about therapy.

## Materials and Methods

### Datasets

For the training set, the RNA-seq transcriptome information and matching clinicopathological data of 502 HNSCC patients were acquired from TCGA (https://portal.gdc.cancer.gov/). For the validation set, 97 patients were retrieved from the Gene Expression Omnibus (GEO; accession number: GSE41613; https://www.ncbi.nlm.nih.gov/geo/query/acc.cgi?acc=GSE41613) (10).

### Immunohistochemistry

Tissue microarray of HNSCC specimens (HOraC080PG01) was obtained from Shanghai Outdo Biotech Company (Shanghai, China) and used to validate the expression of CALR in HNSCC. Immunohistochemistry was then performed using the staining cycles as follows. In brief, formalin-fixed, paraffin-embedded HNSCC tissue sections were deparaffinized and then underwent microwave treatment in citrate for antigen retrieval. Then, they were blocked and incubated overnight with rabbit anti-Calreticulin antibody (1:500, ab92516, Abcam). Goat anti-rabbit IgG (Alexa Fluor 488, ab150077, Abcam) was used as the secondary antibody at 1/1000 dilution. DAPI was used as nuclear counterstain.

### Consensus Clustering

The ConcensusClusterPlus tool in R was utilized to conduct consensus clustering to identify molecular subtypes linked to ICD. Subsequently, we assessed the ideal cluster numbers between k = 2–10, and this process was replicated 1,000 times to guarantee that the results would be stable. The pheatmap tool in R was utilized to create a cluster map.

### Identification of Differentially Expressed Genes (DEGs)

The differential mRNAs expression was assessed utilizing the Limma package (version: 3.40.2) of R software. In order to rectify false-positive TCGA data, the adjusted P values were examined. The screening criteria for mRNAs differential expression determined as adjusted P < 0.05 and | fold change| >2.

### Functional Enrichment Analysis

Gene Ontology (GO) and Kyoto Encyclopedia of Genes and Genomes (KEGG) analyses were carried out to compare the differential signal pathway and biological effects among the ICD low and high cohorts. The ‘‘clusterProfiler’’ package in R software was employed to evaluate GO and KEGG pathways (11). GO and KEGG enrichment analyses were premised on the q-value and p-value thresholds of <0.05.

### Gene Set Enrichment Analysis (GSEA)

GSEA was conducted to assess whether there were considerable variations in the set of genes expressed between the ICD low and high cohorts in the enrichment of the MSigDB Collection (c2.cp.kegg.v7.4.symbols.gmt). The analysis was accomplished using GSEA software (http://www.broadinstitute.org/gsea/index.jsp)

### Characterization of Immune Landscape Between Two ICD Subgroups

To identify immune characteristics of 502 HNSCC samples, their expression data were loaded into CIBERSORT (HTTPS://cibersort.stanford.edu/) and repeated 1000 times to determine the relative percentage of 22 immune cell types (12). Then, we compared the relative percentage of 22 immune cell types between the two ICD subgroups, and the results are presented in a landscape map.

### Prediction of Response to Immunotherapy

Tumor immune dysfunction and exclusion (TIDE) analysis has been performed to determine immunotherapy response. TIDE (http://tide.dfci.harvard.edu/) is an analytic technique which enables a prediction of immunotherapy response using two major tumor immune evasion mechanisms: T cell dysfunction and T cell infiltration inhibited in tumors with low CTL level.

### Somatic Mutation Analysis

Somatic mutation data of the HNSCC samples were obtained from TCGA GDC Data Portal in “maf” format. Waterfall plots were then performed using the “Maftools” package in R software, which facilitated the visualization and summarization of the mutated genes.

### Survival Analysis

Kaplan-Meier (KM) analysis was conducted for comparison of the overall survival (OS) between the low and high ICD risk cohort utilizing the survminer and survival packages in R. The prospective prognostic indicators were identified utilizing the Univariate Cox analysis while the determination of whether the risk score is an independent risk factor for OS in HNSCC was done utilizing the multivariate Cox analysis.

### Construction of the ICD-Related Risk Signature

The immune-associated genes that were found to be statistically significant in the univariate Cox regression analysis were subsequently exposed to a LASSO cox regression analysis to compute the exact coefficient values of each identified association. LASSO is a frequently used regression analysis approach that combines variable selection and regularization to improve the resulting statistical model’s predictive performance and interpretability.

## Results

### Consensus Clustering Identified Two ICD-Associated Subtypes

**Figure 1:**
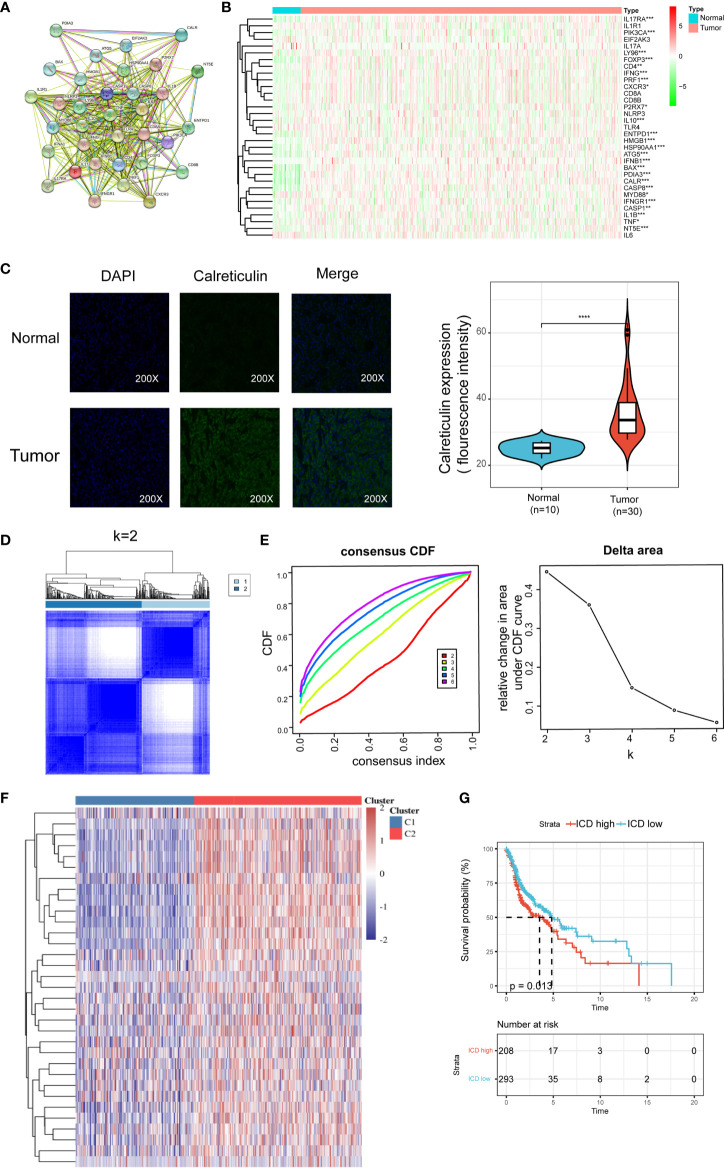
Identification of ICD-associated subtypes by consensus clustering. **(A)** Protein–protein interactions among the ICD-associated genes; **(B)** Heatmap shows 34 ICD gene expression profiles among normal and HNSCC samples in TCGA database; **(C)** Immunohistochemistry validated the expression pattern of CALR in normal and tumor sample; **(D)** Heatmap depicts consensus clustering solution (k = 2) for 36 genes in 502 HNSCC samples; **(E)** Delta area curve of consensus clustering indicates the relative change in area under the cumulative distribution function (CDF) curve for k = 2 to 10; **(F)** Heatmap of 34 ICD-related gene expressions in different subtypes. Red represents high expression and blue represents low expression; **(G)** Kaplan–Meier curves of OS in ICD-high and ICD-low subtypes. *P < 0.05, **P < 0.01, ***P < 0.001, & ****P < 0.0001.

The ICD-related gens were identified by an extensive literature, which has been previously summarized by Abhishek et al (13). We utilized the STRING database to conduct protein-protein interaction (PPI) network analysis to further reveal the connections between these ICD-related genes (**Figure 1A**). We also analyzed the expression patterns of ICD genes in normal and HNSCC samples. Most of ICD genes were overexpressed in HNSCC, including CALR, ENTPD1, NT5E, HMGB1, HSP90AA1, ATG5, BAX, CASP8, PDIA3, PIK3CA, CXCR3, IFNA1, IFNB1, IL10, TNF, CASP1, IL1B, P2RX7, LY96, MYD88, CD4, FOXP3, IFNG, IFNGR1, IL17RA, and PRF1 (**Figure 1B**). We further performed an immunohistochemistry experiment to validate the expression pattern of CALR between tumor and normal samples. And the results show that CALR is overexpressed in HNSCC by IHC (**Figure 1C**).

We next determined the ICD-associated clusters of HNSCC using consensus clustering. Two clusters in the TCGA cohort were identified with distinct ICD genes expression patterns after k-means clustering (**Figures 1D, E**). Overall, clusters C2 showed high ICD-related genes expression levels indicating an ICD-high subtype. On the contrary, clusters C1 presented low expression levels indicating an ICD-low subtype (**Figure 1F**). Therefore, we defined clusters C2 as ICD-high subtype, and clusters C1 as ICD-low subtype. Besides, survival analyses illustrated that these ICD-based subtypes exhibited different clinical results. In general, the ICD-low subtype presented a dismal prognosis and the ICD-high subtype was associated with favorable clinical outcomes (**Figure 1G**).

### Identification of Differentially Expressed Genes (DEGs) and Signal Pathways in Different ICD Subtypes

**Figure 2:**
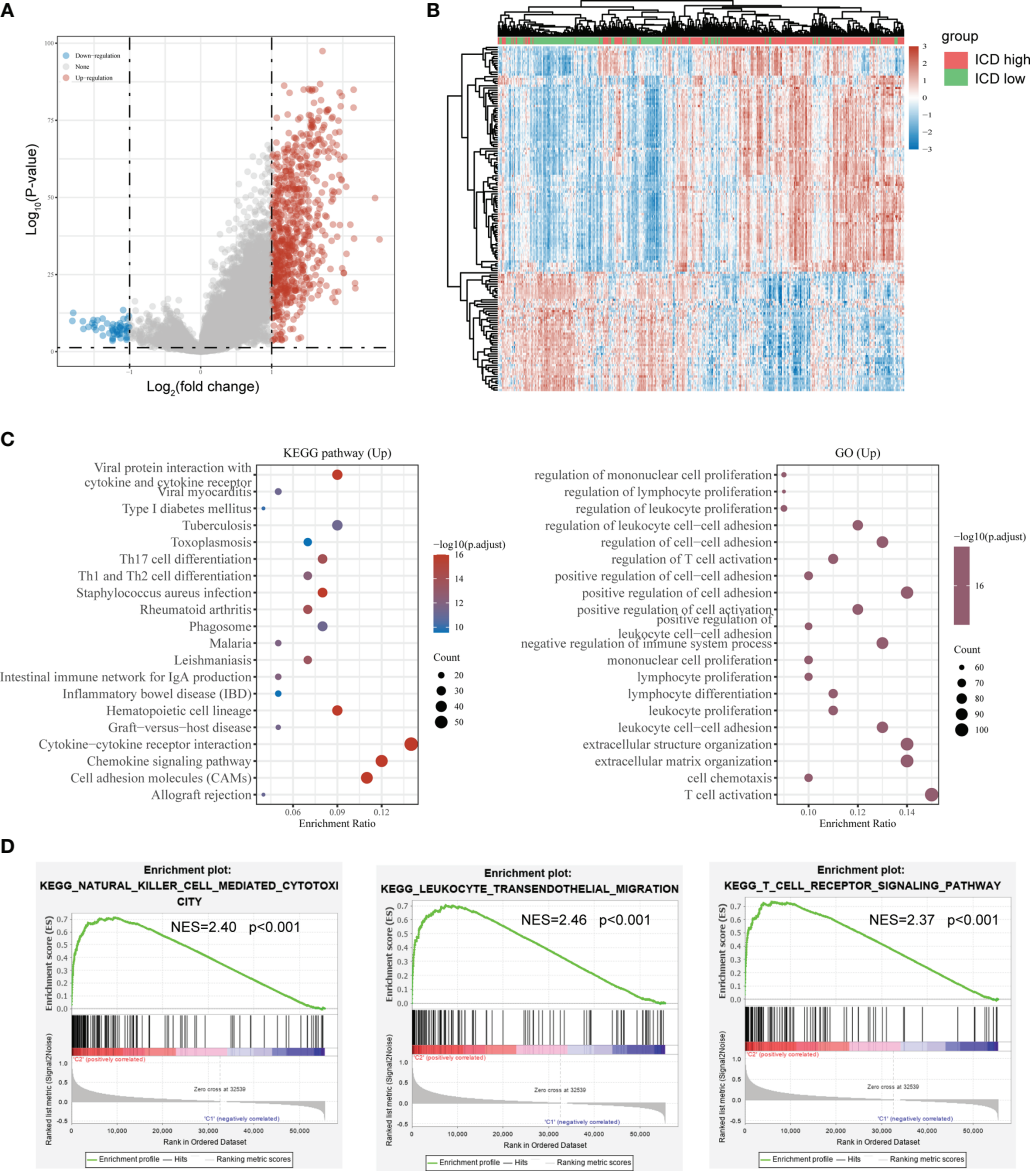
Identification of differentially expressed genes (DEGs) and underlying signal pathways in different subtypes. **(A)** Volcano plot presents the distribution of DEGs quantified between ICD-high and ICD-low subtypes with threshold of |log2 Fold change| > 1 and P < 0.05 in TCGA cohort; **(B)** Heatmap shows the DEG expression in different subtypes; **(C)** Dots plot presents the KEGG and GO signaling pathway enrichment analysis. The size of the dot represents gene count, and the color of the dot represents – log_10_ (p. adjust-value); **(D)** GSEA analysis determines the underlying signal pathway between ICD-high and ICD-low subtypes.

As the ICD high subtype presented with favorable clinical outcomes and the ICD low subtype presented with the dismal prognosis, we identified the key DEGs and signal pathway in each subtype in order to comprehend the molecular mechanism in modulation of prognosis. Here, we found an aggregate of 778 dysregulated genes (**Figures 2A, B**), and the upregulated genes in the ICD high subtype were enriched in activities associated with immunity, including the cytokine and cytokine receptor, Th17 cell differentiation, Th1 and Th2 cell differentiation, regulation of lymphocyte proliferation, regulation of T cell activation, and leukocyte cell−cell adhesion (**Figure 2C**). These results indicated that ICD high subtype was associated with the immune-active microenvironment.

To further identify the associated signaling pathways activated in the ICD high subgroup, we performed GSEA comparing the ICD high and low groups. Gene sets were differentially enriched in the ICD groups, as they were related to immune pathways, such as natural killer cell-mediated cytotoxicity, leukocyte transendothelial migration, and T cell receptor signaling pathway (**Figure 2D**).

### Somatic Mutations and Tumor Microenvironment Landscape in ICD-High and ICD-Low Subtypes

**Figure 3:**
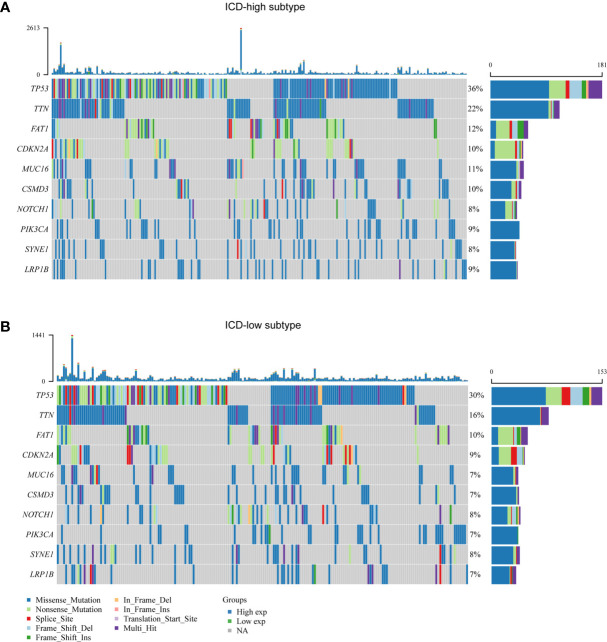
Comparison of somatic mutations between different ICD subtypes. **(A, B)** Oncoprint visualization of the top ten most frequently mutated genes in ICD-high subtype **(A)**, and ICD-low subtype **(B)**.

We noted distinct somatic mutation profiles among these subtypes (**Figures 3A, B**). Despite TP53, TTN, FAT1, CDKN2A and MUC16 were the most frequent mutation, the relative frequencies varied among different subtypes. ICD-high subtypes showed a high frequency of TP53 and TTN mutations, responsible for 36 percent and 22 percent of the total, in that order, while only 30 percent and 16 percent for the ICD-low subtype.

**Figure 4:**
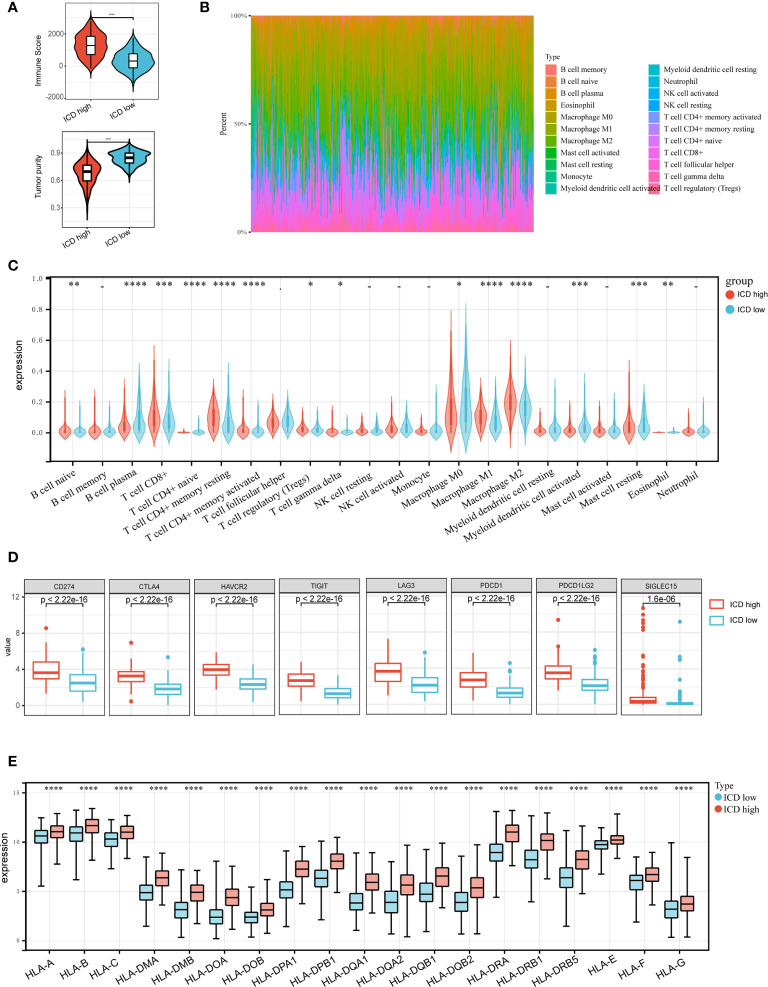
Immune landscape of ICD-high and ICD-low subtypes. **(A)** Violin plots show the median, and quartile estimations for each immune score, and tumor purity score; **(B)** Relative proportion of immune infiltration in ICD-high and ICD-low subtypes; **(C)** Violin plot visualizes significantly different immune cells between different subtypes; **(D, E)** Box plots present differential expression of multiple immune checkpoints **(D)**, and HLA genes **(E)** between ICD-high and ICD-low subtypes. *P < 0.05, **P < 0.01, ***P < 0.001, and ****P < 0.0001.

Accumulating evidence suggests that ICD has a great effect on the activation of certain antitumor immune responses. In this research, we analyzed the composition of the tumor microenvironment between two subtypes. Overall, the immune score was higher and tumor purity was lower in the ICD-high subtype compared to the ICD-low subtype (**Figure 4A**). We next assessed differences in immune infiltration of 22 kinds of immune cells between two subtypes utilizing the CIBERSORT approach in conjunction with the LM22 signature matrix. **Figure 4B** summarizes the results obtained from 502 HNSCC patients in the TCGA. In detail, patients with ICD-high subtype exhibited considerably elevated percentages of B cell plasma, CD8 T cell, resting CD4 T cell memory, activated CD4 T cell memory, T cell regulatory, macrophage M1 and M2, activated myeloid dendritic cell, and eosinophil cell (**Figure 4C**). Besides, most of the human leukocyte antigen (HLA) genes and immune checkpoints were upregulated in the ICD-high subtype. On the contrary, the opposite trend was observed in the ICD-low subtype (**Figures 4D, E**). These indicated that the ICD-high subtype was associated with immune-hot phenotype and the ICD-low subtype was linked to immune-cold phenotype.

### Construction and Validation of the ICD Risk Signature

**Figure 5:**
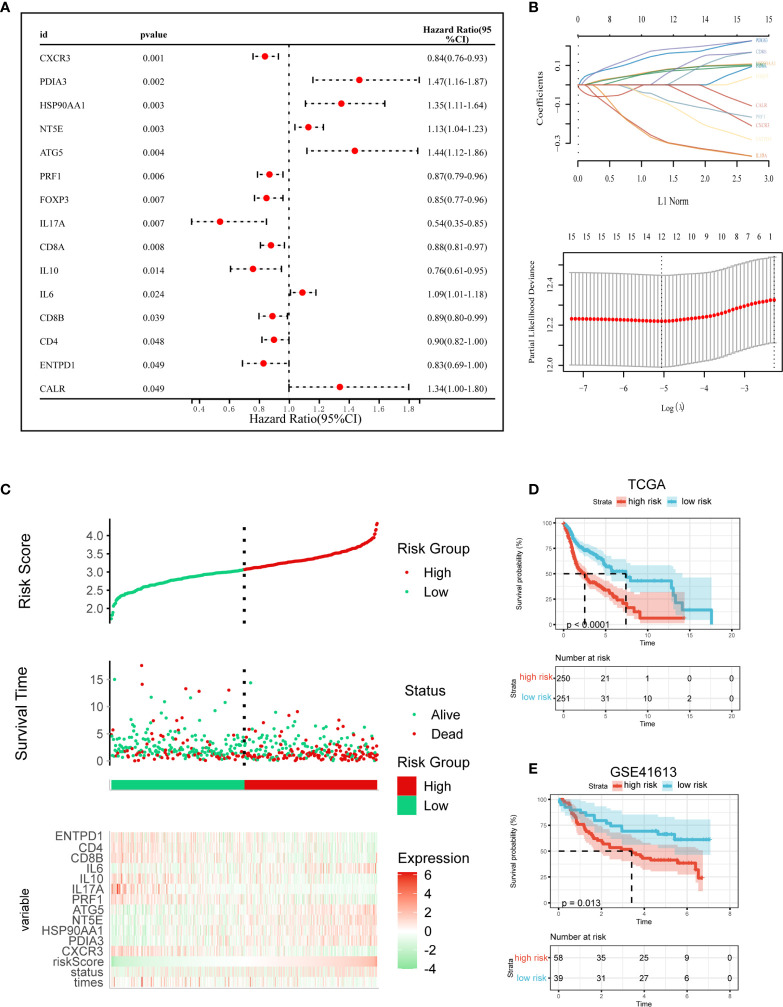
Construction and validation of the ICD risk signature. **(A)** Univariate Cox analysis evaluates the prognostic value of the ICD genes in terms of OS; **(B)** Lasso Cox analysis identified 12 genes most associated with OS in TCGA dataset; **(C)** Risk scores distribution, survival status of each patient, and heatmaps of prognostic 12-gene signature in TCGA database; **(D, E)** Kaplan–Meier analyses demonstrate the prognostic significance of the risk model in TCGA and GSE41613 cohort.

We then created a prognostic model premised on ICD-related genes. 16 ICD-related genes were found to be considerably linked to the OS of patients in the Cox univariate analysis (**Figure 5A**). 12 ICD-related genes were tested and selected for the prediction model in the LASSO regression analysis (**Figure 5B**). The risk-score model was developed premised on the algorithm below: Risk score = (-0.0748)*CXCR3 + (0.1722)*PDIA3 + (0.0945)*HSP90AA1 + (0.0806)*NT5E + (0.1971)*ATG5 + (-0.1156)*PRF1 + (-0.325)* IL17A + (-0.3283)*IL10 + (0.0937)*IL6 + (0.1397)*CD8B + (0.0909)*CD4 + (-0.1915)*ENTPD1. In addition, we investigated the relationship between survival status and risk score. Our results indicated that the number of alive statuses in the low-risk cohort was much greater as opposed to the high-risk cohort (**Figure 5C**). The prognostic significance of this risk profile in HNSCC was further determined utilizing KM analysis (**Figure 5D**). In the TCGA cohort, a high-risk score was found to correspond with poor OS, which was further corroborated by comparable results in the GEO cohort (**Figure 5E**).

### The Association of ICD Risk Signature With Tumor Microenvironment

**Figure 6:**
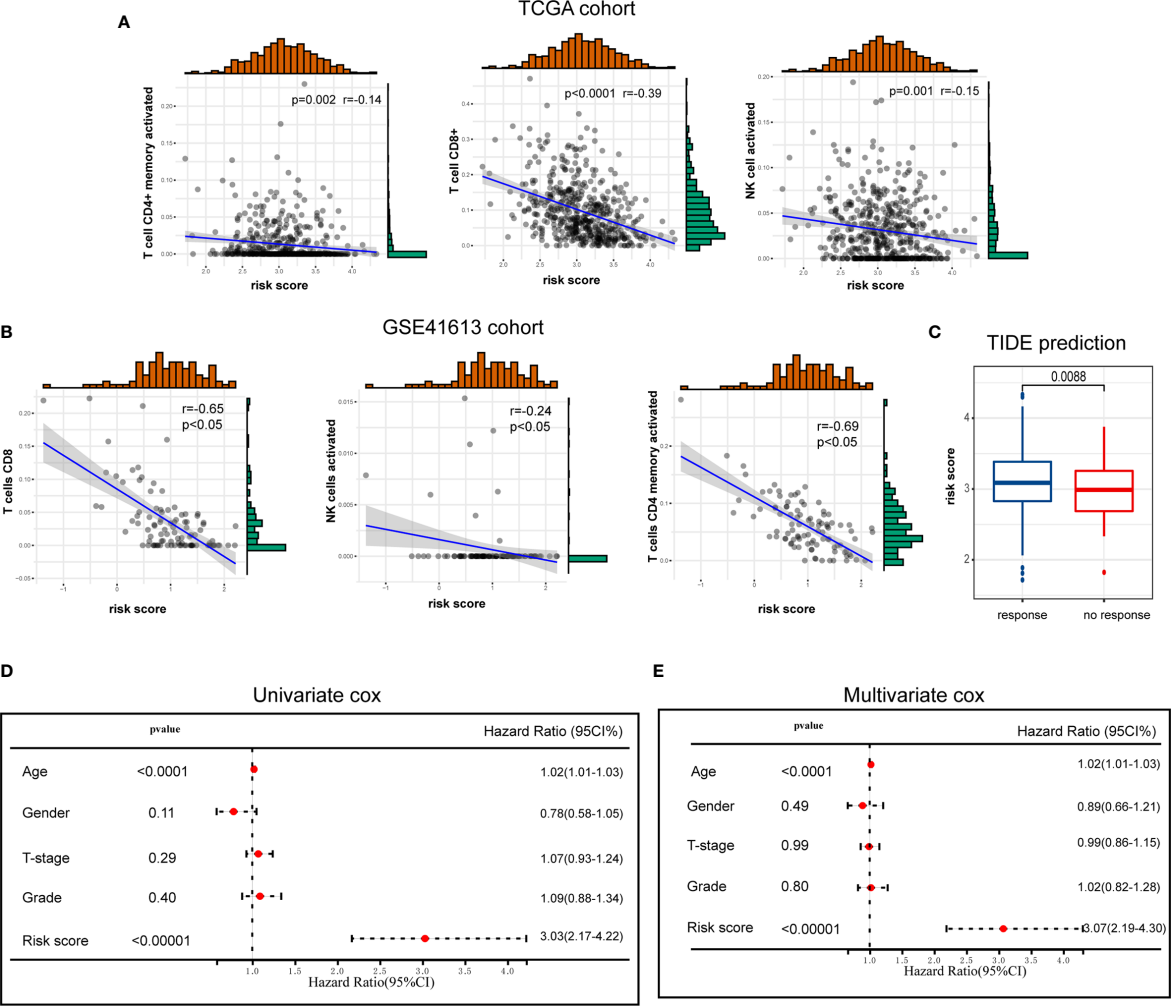
The association of ICD risk signature with tumor microenvironment. **(A, B)** Scatter plots show the correlation of risk score with the infiltration of CD8, activated NK cell, and activated CD4 memory cell **(A)**, which was further validated by GSE41613 cohort **(B)**; **(C)** Box plot presents the association of ICD risk score with immunotherapy response; **(D, E)** Univariate and multivariate Cox analyses evaluate the independent prognostic value of ICD risk signature in HNSCC patients.

Given the significant biological roles of ICD in antitumor immunological responses, the connection between ICD risk score and the tumor microenvironment was thoroughly studied. The results illustrated that patients who exhibited an elevated risk score showed a negative correlation with CD8, activated NK cell, and activated CD4 memory cell (**Figure 6A**). These results were further validated by GEO cohort (**Figure 6B**).

We then used TIDE to evaluate the predictive value of ICD risk signature in the potential clinical efficacy of immunotherapy. In our results, ICD risk scores were higher in immunotherapy no response group, implying that patients with low ICD risk scores could benefit more from immunotherapy (**Figure 6C**).

Univariate and multivariate Cox analyses were performed to evaluate the independent prognostic value of ICD risk signature. The univariate analysis indicated that high ICD risk score was significantly associated with poor OS (**Figure 6D**). Multivariate analysis revealed that ICD risk score could serve as an independent prognostic factor for HNSCC patients (**Figure 6E**).

## Discussion

The notion of immunogenic cell death was described as the unique type of regulated cell death, able to trigger full antigen-specific adaptive immunological responses by emitting danger signals or DAMPs (1, 3, 14). The combination of immunogenic therapeutic and novel immunotherapeutic regimens holds great promise for treating malignancies (15–18). Therefore, it could be advantageous to identify ICD-related biomarkers that help distinguish HNSCC patients premised on the benefits they derive from immunotherapy. Here, we demonstrated that the expressions of ICD-related genes are closely associated with prognosis and tumor microenvironment of HNSCC. We identified two ICD subgroups by consensus clustering based on ICD-related genes expression. ICD high subtype was associated with favorable clinical outcomes and a high level of immune cell infiltration. Besides, we also constructed and validated a prognostic risk signature with 12 selected ICD-related genes, which stratified the HNSCC patients into high- and low-risk cohorts. In addition, this risk signature showed a high predictive value in terms of OS and might function as an independent prognostic indicator for HNSCC patients.

The ICD-related genes analyzed in our study have been previously summarized by Abhishek et al. In brief, ICD parameters were identified by an extensive literature survey (examining Web of Knowledge, Scopus, and PubMed for pertinent research investigations performed *in vivo* using mice and/or *in vitro* using primary human immune cells) (13). Finally, a total of 34 ICD-related genes were extracted and were associated with ovarian, breast, or lung cancer patients’ survival. In our analysis, 15 of the 34 ICD-related genes were considerably linked to the prognosis of HNSCC patients, including CALR, CXCR3, PDIA3, HSP90AA1, NT5E, ATG5, PRF1, FOXP3, IL17A, CD8A, IL10, IL6, CD8B, CD4, and ENTPD1.

Immunogenic cell death (ICD) triggered by cancer therapy reshapes the tumor immune microenvironment (15, 19, 20). Mechanistically, ICD occurs in conjunction with the exposure and release of many DAMPs, which facilitates their interplay with the cognate PRRs exhibited by innate immune cells including DCs, macrophages, and monocytes. This causes these cells to become activated and mature, and they move to drain lymph nodes filled with cancer-derived antigen-specific payloads. The cancer antigens are subsequently exposed to T cells resulting in the enhancement of the immune cell infiltration into the tumor microenvironment (14, 21). In line with this evidence, our study identified two ICD subgroups by consensus clustering, and the ICD-high subgroup was associated with immune-hot phenotype, whereas the ICD-high subgroup was referred to as immune-cold phenotype.

In conclusion, our study highlights the associations of the ICD subtypes with changes in the immunological tumor microenvironment in HNSCC. These observations may benefit the immune therapy-based interventions for HNSCC patients. We also constructed and validated an ICD-related prognostic signature, which proved significant value in predicting OS time of HNSCC patients.

## Data Availability Statement

Publicly available datasets were analyzed in this study. This data can be found here: https://portal.gdc.cancer.gov/ (TCGA-HNSC) and https://www.ncbi.nlm.nih.gov/geo/query/acc.cgi?acc=GSE41613.

## Ethics Statement

The studies involving human participants were reviewed and approved by Ethics Review Committee of the Shanghai Outdo Biotech Company. Written informed consent for participation was not required for this study in accordance with the national legislation and the institutional requirements.

## Author Contributions

XinwenW and SW: methodology, and writing. FL and DK: software. XinwuW, DP, and WX: validation. LZ and WH: data curation, and conceptualization. All authors contributed to the article and approved the submitted version.

## Funding

This work was supported by Jiangmen Medical and Health Technology Plan Project (Project No. 2019B003).

## Conflict of Interest

The authors declare that the research was conducted in the absence of any commercial or financial relationships that could be construed as a potential conflict of interest.

## Publisher’s Note

All claims expressed in this article are solely those of the authors and do not necessarily represent those of their affiliated organizations, or those of the publisher, the editors and the reviewers. Any product that may be evaluated in this article, or claim that may be made by its manufacturer, is not guaranteed or endorsed by the publisher.
